# 晚期肺腺癌患者化疗前后血清*EGFR*基因突变状态的比较

**DOI:** 10.3779/j.issn.1009-3419.2011.02.04

**Published:** 2011-02-20

**Authors:** 如冰 韩, 巍 钟, 静 赵, 力 张, 莹 夏, 寒 王, 龙芸 李, 孟昭 王

**Affiliations:** 100730 北京，中国医学科学院，北京协和医学院，北京协和医院呼吸科 Department of Respiratory Disease, Peking Union Medical College Hospital, Peking Union Medical College, Chinese Academy of Medical Sciences, Beijing 100730, China

**Keywords:** 肺肿瘤, 突变, 化疗, Lung neoplasms, Mutation, Chemotherapy

## Abstract

**背景与目的:**

存在表皮生长因子受体（epidermal growth factor receptor, EGFR）基因突变的非小细胞肺癌（non-small cell lung cancer, NSCLC）作为NSCLC的一个特殊亚群，对于表皮生长因子酪氨酸激酶抑制剂（epidermal growth factor receptor tyrosine kinase inhibitor, EGFR-TKI）的治疗显示出良好的疗效。本研究旨在检测晚期肺腺癌患者化疗前后血清*EGFR*基因外显子19和外显子21的突变状态，并分析化疗是否对*EGFR*基因突变状态产生影响。

**方法:**

磁珠法提取血清游离DNA后，使用酶切富集巢式PCR分别对血清游离DNA中*EGFR*外显子19和外显子21进行特异性扩增，应用直接测序法对*EGFR*基因突变状态进行检测。

**结果:**

33例肺腺癌患者化疗前*EGFR*基因突变率为39.4%（13/33），化疗后为54.5%（18/33），化疗前后*EGFR*基因突变状态的一致率为54.5%（18/33）；在不一致的15例患者中，10例由化疗前*EGFR*基因突变阴性变为阳性，5例由化疗前阳性变为阴性。

**结论:**

化疗可能导致血清*EGFR*基因突变状态的改变。

## Abstract (en)

**Background and objective:**

Non-small cell lung cancer (NSCLC) with mutations in the epidermal growth factor receptor (EGFR) is a distinct subgroup of NSCLC, which is particularly responsive to EGFR tyrosine kinase inhibitors (TKIs). The aim of this study is to detect *EGFR* mutations in paired serum of pre- and post-chemotherapy from advanced pulmonary adenocarcinoma patients to evaluate impact of chemotherapy on *EGFR* mutation status.

**Methods:**

Magnetic beads were used for DNA extraction from paired serum of pre- and post-chemotherapy of 33 advanced pulmonary adenocarcinoma patients. The *EGFR* exon 19 and 21 were amplified by mutant-enriched nested PCR and analyzed by direct sequencing.

**Results:**

*EGFR* mutations were detected in 39.4% (13/33) and 54.5% (18/33) serum samples of pre- and postchemotherapy, respectively. The *EGFR* mutation status was consistent in 54.5% (18/33) patients. Among 15 discordant cases, 10 changed from pre-chemo wild-type to post-chemo mutant-type status, while 5 from pre-chemo mutant-type to post-chemo wild-type status.

**Conclusion:**

Chemotherapy may have influence on serum *EGFR* mutation status in advanced adenocacinoma patients.

越来越多的证据表明非小细胞肺癌(non-small cell lung cancer, NSCLC)表皮生长因子受体(epidermal growth factor receptor, EGFR)基因突变状态影响NSCLC的治疗策略。近90%的*EGFR*基因突变集中于外显子19的缺失突变和外显子21点突变^[1, 2]^。对于*EGFR*基因突变阳性的NSCLC患者，EGFR酪氨酸激酶抑制剂(epidermal growth factor receptor tyrosine kinase inhibitor, EGFR-TKI)，包括吉非替尼和厄洛替尼，有明显疗效。然而我们应注意到，对于最初*EGFR*基因突变阳性的人群，EGFR-TKIs作为二/三线治疗和一线治疗的客观有效率是有差异的，分别为30%-40%^[3-5]^和70%^[6-8]^。因此我们推测化疗会对*EGFR*基因突变状态产生影响，从而影响EGFR-TKIs作为NSCLC二/三线治疗的有效率。

然而化疗前后分别取肺癌组织标本进行*EGFR*基因突变检测临床实践困难。研究^[9]^表明，实体肿瘤形成和发展过程中常有少量的肿瘤细胞从原发肿瘤脱落进入血循环，此类癌细胞在血循环中凋亡并将遗传物质释放到血液中，这提示血液游离DNA可成为检测肿瘤基因突变的一种方法，而且标本获得更加方便。几项较大样本的研究^[10, 11]^均发现晚期肺癌患者血清和肿瘤组织*EGFR*基因突变状态检测的一致性为79.7%-94%，这些研究提示血清DNA可替代肿瘤组织DNA进行肿瘤*EGFR*基因突变检测。本研究收集晚期肺腺癌患者化疗前后的配对血清标本，使用酶切突变富集巢式PCR法直接测序进行*EGFR*基因突变检测，观察化疗对*EGFR*基因突变状态是否存在影响。

## 1 资料与方法

### 1.1 研究对象

收集2009年11月-2010年5月就诊于北京协和医院呼吸内科肺癌中心的患者血清标本。患者入选标准包括：组织学和/或细胞学证实的肺腺癌患者，分期为Ⅲb期或Ⅳ期，有全身化疗前和化疗2个-6个周期后的配对标本。每次收集全血标本2 mL，分离血清，-20 ℃保存。共收集化疗前后配对标本33例。所有患者均签署知情同意书。

### 1.2 实验方法

采用磁珠法提取200 μL血清中游离DNA (磁珠法游离DNA提取试剂盒，北京金麦格生物技术有限公司)，获得游离DNA 30 μL，测得平均DNA浓度为7.3 ng/μL(2.7 ng/μL-17.4 ng/μL)，置于1.5 mL离心管中冻存备用。

*EGFR*基因外显子19和21的扩增采用酶切突变富集巢式PCR法。引物的序列见表 1(上海Invitrogen公司合成)。第一轮PCR反应体系为10 μL，包括GoTaq mix 5 μL、上下游引物(10 pm/μL)各0.5 μL、基因组DNA 3 μL，无核酸水补齐至10 μL。反应条件为95 ℃、5 min；95 ℃、30 s，56 ℃、30 s，72 ℃、50 s，5个循环；95 ℃、3 s，61 ℃、30 s，72 ℃、50 s，15个循环；72 ℃、10 min。

**1:** *EGFR*基因外显子19和21巢式PCR扩增引物序列 Primer sequences used for nested PCR of exon 19 and 21 of *EGFR* gene

Exon	Primer	Primer sequences	Fragment
Exon 19	Outer	Forward: 5'-CCAGATCACTGGGCAGCATGTGGCACC-3'	265 bp
		Inverse: 5'-AGCAGGGTCTAGAGCAGCAGCTGCC-3'	
	Inner	Forward: 5'-CGTCTTCCTTCTCTCTCTGTCAT-3'	148 bp
		Inverse: 5'-CCACACAGCAAAGCAGAAAC-3'	
Exon 21	Outer	Forward: 5'-TCAGAGCCTGGCATGAACATGACCCTG-3'	297 bp
		Inverse: 5'-GGTCCCTGGTGTCAGGAAAATGCTGG-3'	
	Inner	Forward: 5'-CAGCAGGGTCTTCTCTGTTTC-3'	213 bp
		Inverse: 5'-GAAAATGCTGGCTGACCTAAAG-3'	

酶切反应体系及条件：体系20 μL，包括PCR1产物1 μL、限制性内切酶Mse Ⅰ 0.5 μL(外显子19)或限制性内切酶Msc Ⅰ 0.5 μL(外显子21)、10×buffer 1 μL，无核酸水补齐至20 μL；反应条件为37 ℃、5 h。

第二轮PCR反应体系为25 μL，包括GoTaq mix 12.5 μL、上下游引物(10 pm/μL)各1.5 μL、模板DNA(即PCR1产物酶切后的DNA)1 μL，无核酸水补齐至25 μL。外显子19反应条件为95 ℃、5 min；95 ℃、30 s，55 ℃、30 s，72 ℃、30 s，40个循环；72 ℃、10 min。外显子21反应条件为95 ℃、5 min；95 ℃、30 s，58 ℃、30 s，72 ℃、30 s，40个循环；72 ℃、10 min。

### 1.3 *EGFR*基因外显子19和21突变状态判断

最终PCR产物由上海Invitrogen公司完成测序，使用DNAMAN软件阅读序列。*EGFR*基因外显子19突变为缺失突变，巢式PCR扩增产物序列测序结果与野生序列进行比对，若与野生型序列吻合，判读为野生型；若为单一序列且其序列表现为一段碱基缺失时，判读为突变型；若为野生型序列和突变型序列的叠加，也判读为突变型(图 1)。*EGFR*基因外显子21突变为L858R点突变，巢式PCR扩增产物序列测序结果与野生序列进行比对，若与野生型序列吻合，判读为野生型；若为单一序列且其序列表现为突变型碱基G时，判读为突变型；若为野生型序列和突变型序列的叠加，即野生型碱基T和突变型碱基G混合，也判读为突变型(图 2)。*EGFR*基因敏感突变包括外显子19的缺失突变和/或外显子的21点突变。

**1:**
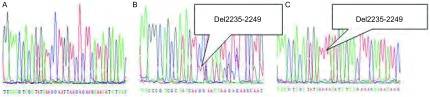
*EGFR*外显子19 PCR2产物直接测序图。A：野生型序列；B：混合型序列；C：突变型序列。 Direct sequencing of PCR2 products for exon 19 of *EGFR* gene. A: wild type; B: mixture of both wild and mutant type; C: mutant type.

**2:**
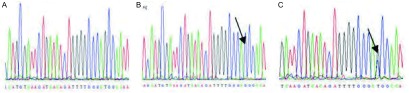
*EGFR*外显子21 PCR产物直接测序图。A：野生型序列；B：混合型序列；C：突变型序列。 Direct sequencing of PCR products for exon 21 of *EGFR* gene. A: wild type; B: mixture of both wild and mutant type; C: mutant type.

### 1.4 统计学分析

使用SPSS 16.0软件进行统计分析，使用χ^2^检验分析不同亚组患者*EGFR*基因突变率的差异，*P* < 0.05为差异有统计学意义。

## 2 结果

### 2.1 患者的一般情况

入组33例患者中男性20例，女性13例；年龄36岁-75岁，中位年龄57岁；吸烟者11例，不吸烟者22例。其中29例接受一线化疗，方案包括吉西他滨联合铂类13例，紫杉醇联合铂类10例，培美曲塞联合顺铂5例，VP-16联合顺铂1例；4例接受二线单药多西紫杉醇化疗。化疗疗效：部分缓解(partial response, PR)5例，疾病稳定(stable disease, SD)22例，疾病进展(progresive disease, PD)6例。

### 2.2 化疗前血清*EGFR*基因突变状态

化疗前血清DNA样本检测结果显示患者*EGFR*基因突变发生率为3 9. 4%(1 3 / 3 3)。外显子1 9突变8个，突变率为24.2%(8/33)，外显子21突变11个，突变率为33.3% (11/33)，可见以外显子21突变为主(57.9%, 11/19)，其中6例患者同时伴有外显子19和21突变。女性患者*EGFR*基因突变率为46.2%(6/13)，男性患者突变率为35.0%(7/20)，女性高于男性，但无统计学差异(*P*=0.52)。吸烟患者突变率为45.5%(5/11)，不吸烟患者突变率为36.4%(8/22)，吸烟患者略高于不吸烟患者，但无统计学差异(*P*=0.61)。

### 2.3 化疗后血清*EGFR*基因突变状态

化疗后血清DNA样本检测结果显示患者*EGFR*基因突变发生率为54.5%(18/33)。外显子19突变10个，突变率为30.3%(10/33)，外显子21突变14个，突变率42.4% (14/33)，可见以外显子21突变为主(58.3%, 14/24)，其中6例患者同时伴有外显子19和21突变。女性患者*EGFR*基因突变率为38.5%(5/13)，男性患者突变率为65.0%(13/20)，女性低于男性，但无统计学差异(*P*=0.14)。吸烟患者突变率为72.8%(8/11)，不吸烟患者突变率为45.5%(10/22)，吸烟患者高于不吸烟患者，但无统计学差异(*P*=0.14)。

### 2.4 化疗前后的比较

化疗前患者检出*EGFR*基因敏感突变阳性率为39.4%(13/33)，化疗后患者检出*EGFR*基因敏感突变阳性率为54.5%(18/33)，无统计学差异(*P*=0.51)。患者化疗前后*EGFR*基因敏感突变状态一致率为54.5%(18/33)，患者化疗前后*EGFR*基因敏感突变具体变化见表 2。

**2:** 33例患者化疗前后*EGFR*基因突变状态具体变化 The *EGFR* gene mutation status of pre- and post-chemotherapy serum samples in 33 patients enrolled

		*EGFR* mutation status of post-chemotherapy mutation status of post-chemotherapy		Total
		Negtive	Positive	
*EGFR* mutation status of	Negative	10	10	20
Pre-chemotherapy	Positive	5	8	13
Total		15	18	33

对于EGFR外显子19缺失突变，化疗前阳性率为24.2%(8/33)，化疗后阳性率为30.3%(10/33)，无统计学差异(*P*=0.16)，其中化疗前后均阳性4例，化疗前后均阴性19例患者，化疗前阴性化疗后阳性6例，化疗前阳性化疗后阴性4例。对于EGFR外显子21点突变，化疗前阳性率为33.3%(11/33)，化疗后阳性率为42.4%(14/33)，无统计学差异(*P*=0.08)，其中化疗前后均阳性7例，化疗前后均阴性15例，化疗前阴性化疗后阳性7例，化疗前阳性化疗后阴性4例。

### 2.5 化疗前后*EGFR*基因突变状态变化与疗效的关系

化疗前后*EGFR*基因突变状态变化与疗效的关系见表 3，可见基因突变状态的变化与化疗疗效无明显相关性(χ^2^=3.26, *P*=0.775)。

**3:** 33例患者化疗前后*EGFR*基因突变状态变化与疗效的关系 The profile of *EGFR* gene mutation status of pre- and post-chemotherapy serum samples and its relation with efficacy of chemotherapy in 33 patients enrolled

Changes of *EGFR* mutation status of pre- and post-chemotherapy	Efficacy evaluation		
	PR	SD	PD
Negative→Negative	1 (20%)	7 (31.8%)	2 (33.3%)
Positive→Negative	0 (0%)	4 (18.2%)	1 (16.7%)
Negative→Positive	3 (60%)	6 (27.3%)	1 (16.7%)
Positive→Positive	1 (20%)	5 (22.7%)	2 (33.3%)
Total	5	22	6
PR: partial response; SD: stable disease; PD: progressive disease.			

## 3 讨论

本研究前瞻性分析了化疗是否会对NSCLC患者*EGFR*基因突变状态产生影响，结果显示化疗后*EGFR*基因敏感突变阳性率似乎高于化疗前(54.5% *vs* 39.4%)，化疗前后*EGFR*基因突变状态一致率仅为54.5% (18/33)；不一致的15例中，10例由化疗前*EGFR*基因突变阴性变为阳性，5例由化疗前阳性变为阴性。

分析化疗前后*EGFR*基因突变状态发生变化的原因有很多推测或假设。首先，化疗对于*EGFR*基因突变阳性的肿瘤细胞表现出更强的活性，可以使外周血中*EGFR*突变基因比例增高。IPASS研究^[7]^中EGFR基因突变阳性人群化疗的客观有效率为47.3%；日本NEJ002研究^[8]^中*EGFR*基因突变阳性人群一线化疗的客观有效率为29%；WJTOG3405研究^[12]^中EGFR基因突变阳性人群一线化疗的客观有效率为32.2%，均高于文献报道的非选择性人群含铂双药方案的大约20%的客观有效率，这提示*EGFR*基因突变阳性肿瘤对化疗药物更加敏感，化疗可能使*EGFR*基因突变阳性的肿瘤细胞更易被杀伤，因而释放更多DNA入血，因此化疗后突变阳性比例升高。

其次，肿瘤在进展过程中*EGFR*基因突变状态可能发生变化。Gow等^[13]^对67例NSCLC患者肿瘤组织及其相应的转移灶进行EGFR基因突变检测，结果显示18例原发灶样品*EGFR*基因突变阳性，而其中9例(50%)转移灶表现为*EGFR*基因突变阴性，而转移灶表现为*EGFR*基因突变阳性的26例样品中，相应的原发灶有17例(65%)表现为突变阴性，原发灶与转移灶*EGFR*基因突变状态的不一致多是由于转移灶表现为*EGFR*基因突变阳性而原发灶表现为阴性造成的。因此，在肿瘤发展过程中可能存在*EGFR*基因突变状态的改变，而且可能以转移灶*EGFR*基因突变阳性率增高为主。

化疗也可能促使*EGFR*基因耐药突变的产生。Chin等^[14]^将含有EGFR外显子19缺失突变的NSCLC细胞系-PC9细胞系经过铂类化疗药物处理耐药后，再使用厄洛替尼，发现厄洛替尼的敏感性降低，说明化疗可能在酪氨酸激酶激活通路中发挥作用。因此二/三线EGFR-TKIs治疗有效率的降低可能部分归因于化疗导致的EGFR-TKIs耐药，具体机制有待进一步临床研究证实。

本研究结果提示化疗前后肺腺癌患者的*EGFR*基因突变状态可能会发生变化，本研究虽然采用了前瞻性设计来收集标本，但是仍然存在一定缺陷。样本量小会产生一定的偏差，需要设计大样本多中心的临床试验；采用外周血DNA检测，虽然样本能相对容易获得，但不如组织标本更有说服力，需要化疗前后的患者组织标本来证实我们发现的现象；本实验中外周血标本是在化疗前和化疗后采集的，实际上应该采集化疗前和化疗后患者疾病进展时的标本进行检测，更能对治疗提供线索。因此希望能开展多中心大样本的临床试验并收集化疗前和化疗后疾病进展时的组织标本进行检测以证实化疗对EGFR基因突变状态的影响。
